# The Effectiveness and efficiency of corporate innovation under strict market regulation: Based on China’s new securities law

**DOI:** 10.1371/journal.pone.0326110

**Published:** 2025-07-23

**Authors:** Zhenna Huang

**Affiliations:** Faculty of Finance, City University of Macau, Macau, China; South China Normal University School of Economics and Management, CHINA

## Abstract

The revision of the new securities law (NSL) represents a market-oriented reform of China’s securities market centred on the registration-based system. On the basis of data from all A-share listed companies in China from 2015 to 2022, this study employs the NSL as a quasinatural experiment and uses the difference-in-differences (DID) method to evaluate the impact of NSL implementation on the effectiveness and efficiency of corporate innovation. The empirical results indicate that the implementation of the NSL has a significant positive effect on both the effectiveness and efficiency of corporate innovation. This conclusion remains robust after supplementary tests, such as changing the regression method, replacing the dependent variables, excluding municipalities directly under the central government, and accounting for the impact of the pandemic, are conducted. The mediation effect analysis reveals that NSL implementation significantly affects corporate innovation by alleviating financing constraints, enhancing firms’ risk-taking capacity, and strengthening corporate governance. Furthermore, the moderation effect analysis indicates that the corporate governance environment influences the effectiveness of the NSL on corporate innovation. In a poor corporate governance environment, the benefits of the NSL may not effectively translate into improved corporate innovation outcomes. This study provides important evidence for advancing capital market reforms and improving corporate governance while emphasizing the policy implications of strengthening legal protections to enhance corporate innovation capabilities.

## 1. Introduction

The new securities law (NSL) came into effect on 1 March 2020 and its implementation has played a key role in regulating market order, protecting investors’ rights and interests, promoting the healthy development of the market, and enhancing market efficiency. Its core functions can be summarized as follows: implementing a comprehensive reform of the registration system; shifting China’s stock market from an approval mechanism to a registration system; strengthening the penalties for noncompliance; enhancing legal obligations; increasing fines for stock trading violations; supporting the rule of law; emphasizing the transparency of information disclosure; optimizing investor protection measures; expanding the types of listed companies; expanding the number of companies listed in the market according to the type of investor (including public investors and professional investors); and increasing the number of investors in the market. The level of corporate innovation usually refers to the extent to which companies have innovated in the past, the extent to which they have innovated in the present, the extent to which they have innovated in the past, and the extent to which they have innovated in the future. The level of corporate innovation usually refers to the comprehensive manifestation of a company’s innovation ability and innovation results in terms of products, services, technologies, management, business models, markets, cultures, organizations, processes, and sustainability. Therefore, it is important to study how the strict regulation of the capital market brought about by the NSL will affect the level of corporate innovation through what kind of mechanism, which is highly important for the value creation of Chinese enterprises, the development of the capital market and the high-quality development of the economy.

Existing research on the NSL predominantly examines its impact on audit pricing and market regulation, as well as legal proceedings and violations. Studies show that the NSL significantly increases audit fees due to heightened disciplinary risks and reduced market concentration, with these effects amplified by increased transparency and strengthened investor protections [1,2]. Case studies have highlighted the shift in regulatory practices, such as the “zero tolerance” approach observed in the Shanghai Yingyi case, which underscores the deterrent strength of the NSL and its alignment with responsive regulation principles [3]. The law’s dual-track system has improved investor protection and enhanced market liquidity by reducing bond credit spreads and mitigating default risks [4]. Furthermore, the NSL strengthens the precision of analyst forecasts and reduces corporate tax avoidance by enhancing information quality and legal deterrence [5]. However, challenges remain in defining and addressing market manipulation, as existing legal frameworks require further refinement to align with the complexities of regulatory practices [6].

Empirical research on the NSL has focused primarily on its role in strengthening legal frameworks, mandating information disclosure, and protecting small and medium–sized investors’ rights. Violations in disclosure have been shown to harm investor interests, emphasizing the need for robust legal protections [7]. Studies have also explored the relationships between corporate innovation and various factors, such as management influence, shareholder protections, and financial incentives. For instance, Ning et al. found that CEOs with strong family cultural imprints often hinder innovation [8], whereas tax incentives and strengthened legal protections improve innovation by reducing financial constraints and enhancing resource allocation [9,10]. Additionally, increasing human capital and reducing information asymmetry in the financial sector have been identified as crucial drivers of innovation [11]. These findings underscore the importance of aligning governance mechanisms, financial incentives, and investor protections to foster corporate innovation effectively.

The implementation of China’s NSL in 2020 marked a significant shift towards a market-oriented securities system by replacing the approval-based mechanism with a registration-based approach. This reform aimed to enhance market transparency, strengthen investor protections, and improve corporate governance. While existing studies have explored the NSL’s impact on audit pricing, legal deterrence, and market manipulation [1,3], limited attention has been given to how the NSL influences corporate innovation. Specifically, there is a lack of research on the mechanisms through which the NSL affects innovation effectiveness and efficiency, such as by alleviating financial constraints and enhancing risk-taking capabilities. Furthermore, little is known about the heterogeneity of these effects across different firm characteristics, including ownership structure, lifecycle stage, and market competition intensity.

This study addresses these gaps by employing a difference-in-differences (DID) model approach to evaluate the NSL’s impact on corporate innovation in China. Using a comprehensive dataset of A-share listed firms from 2015 to 2022, this study examines how the NSL improves innovation outcomes through financial and governance mechanisms and investigates its heterogeneous effects across firms. The findings contribute to the literature on corporate innovation and legal reforms by offering new insights into how regulatory changes promote innovation in a strongly regulated capital market.

## 2. Policy background and theoretical analysis

### 2.1 Policy background

China’s inaugural Securities Law was promulgated on 29 December 1998 and was scheduled to take effect on 1 July 1999 the following year. The legislation has been revised or amended on several occasions by the legislature, the Standing Committee of the National People’s Congress (NPC), in accordance with the country’s economic development and changes in the financial market. On 28 December 2019, the 15th meeting of the Standing Committee of the 13th NPC adopted the latest revised version of the legislation, which stipulated that it would come into effect in the spring of March 2020. The legislation came into force in the spring of March 2020 and was implemented in the following year.

In 2019, the promulgation of the NSL marked the entry of China’s capital market into the era of “harsh and severe laws”. Specifically, with respect to administrative liability, the maximum amount of administrative penalties was significantly increased. Previously, the majority of administrative penalties imposed for corporate violations were capped at 600,000 RMB. However, the latest version of the legislation increased this amount to 10 million RMB while also significantly increasing the cap on individual penalties. In terms of civil liability, a representative action system (class action system) was introduced, enabling the selection of a representative in the event of many parties. Judicial recourse brought by organizations that ensure investors’ rights and interests is based on the principle of “default entry without express withdrawal”. In July 2020, the Supreme Court promulgated a rule entitled “Some Issues Relating to Representative Litigation in Securities Disputes” to increase the efficiency of the class action litigation process. The new regulation removes the requirement for registration in the case of noncontentious representative cases and significantly simplifies the conditions for initiating legal proceedings in contentious representative cases.

### 2.2 Theoretical analysis and research hypotheses

After the enactment of the NSL, the impact of digital finance on improving the efficiency of corporate capital markets became more pronounced, thus effectively mitigating stock price synchronization [12]. The government has implemented targeted policies to promote the development of digital finance and amplify its beneficial effects on corporate capital market efficiency [12]. While upgrading the quality of STI firms, optimizing financial management regulations, and enhancing the guidance of fiscal and tax strategies, we also need to actively pursue changes in the pricing system, trading system, and exit process to achieve a rational allocation of capital to STI firms, thus generating more robust and innovative investments [13]. Capital markets play a key role in facilitating the convergence of technology and capital and in establishing an institutional framework for the development of efficient innovation financing models and sustained liquidity [13]. Since the beginning of 2019, the pace of China’s capital market reform has accelerated significantly. This includes the launch of the Science and Technology Innovation Board, the adjustment of the registration system of the Growth Enterprise Market, the implementation of the NSL, and the implementation of the new policy on delisting, which have significantly accelerated the progress of capital market marketization, globalization, and legalization [13]. However, sustained efforts are required to enhance capital market standards and reinforce policy guidance, thereby directing capital flows towards STI enterprises and fostering healthy and sustainable economic expansion [13].

Furthermore, the NSL’s regulatory framework has implications for market dynamics, influencing investor sentiment and stock market behaviour, which, in turn, affect corporate innovation incentives. Kandasamy and Bechkoum showed that social media sentiment can significantly affect stock market volatility, a phenomenon that could shape investor expectations and influence corporate decision-making [14]. As the NSL improves information transparency and investor protections, it enhances the reliability of market signals, potentially stabilizing market conditions and providing a more predictable environment for long-term innovation investments. The relationship between corporate innovation and institutional frameworks is further underscored by Akbar et al. [15]. Strong institutional environments foster innovation by ensuring legal protections and financial market efficiency [16]. The NSL, by enhancing legal safeguards and market access, contributes to this environment, encouraging firms to invest in innovative projects with greater confidence.

There is a paucity of literature examining the impact of the implementation of the NSL on the effectiveness and efficiency of corporate innovation under the strong regulation of the capital market. Therefore, the author proposes the following hypothesis.

H1: The implementation of the NSL under the strong regulation of the capital market has a significant positive effect on the issue of corporate innovation effectiveness and efficacy.

The introduction of the new capital management regulations has been demonstrated to enhance firms’ innovation performance. The impact of the new rules on capital management on enterprises’ R&D investment is more pronounced in contexts where enterprise financing constraints are less pronounced [17]. Financial leasing has a positive effect on the innovation input and output of enterprises after they obtain funds through financial leasing. Following the acquisition of funds through financial leasing, the constraints associated with enterprise financing can be alleviated, thereby facilitating an increase in R&D input and innovation output [18]. The degree of openness in the capital market exerts a regulatory influence on the technological innovation of distribution enterprises, operating through the mechanism of the effect of financial resources [19]. The development of a high-level open capital market is accompanied by the emergence of a more transparent information disclosure mechanism and a more perfect market supervision system. In such a market, circulation enterprises can obtain more accurate financing information with lower information search costs, enabling them to secure financing within a more reasonable range, which provides more accurate capital financing services for the technological innovation of circulation enterprises and is conducive to the continuity and dynamics of technological innovation activities [19]. This is conducive to the continuity and dynamics of technological innovation activities [19]. Conversely, opening the capital market mitigates the risk of technological innovation in distribution enterprises by alleviating financing constraints [19]. In addition to opening the capital market, external investors increase the technological innovation of distribution enterprises, so high-quality information disclosure is not only conducive to a better understanding of the operating conditions of the enterprise by investors but also helps reduce the risk of technological innovation and improve the success rate of technological innovation through the integration and analysis of market information and nonstandard information faced by professional investment research institutions [19]. The risk tolerance of publicly listed firms is not the same as the risk tolerance of their publicly listed counterparts. There is a strong relationship between the risk tolerance of publicly traded firms and their innovativeness. The greater the stress resistance and risk tolerance of senior managers are, the more conducive they are to enhancing the firm’s risk tolerance. Increased investment in innovation programs can help firms improve their innovative strength [20].

H2: The implementation of the NSL under the strong regulation of the capital market significantly improves the effectiveness and efficacy of corporate innovation by easing financing restrictions and enhancing corporate risk-bearing capacity.

The implementation of the Shanghai–Shenzhen–Hong Kong Stock Connect policy has led to notable increases in firms’ innovation inputs, outputs, and efficiencies. This process is significantly influenced by the corporate governance mechanism [21]. From the perspective of incentive channels, the CSI policy can improve the equity incentives and compensation incentives for executives, improve the innovation ability of the executive team, and increase the government subsidies that enterprises can obtain. This fully mobilizes the enthusiasm of enterprises to engage in innovation activities. From the perspective of monitoring channels, the CSI policy can reinforce the monitoring of controlling shareholders’ equity pledge behaviour and managers’ agency behaviour. Furthermore, it can attract additional analysts and investors who are attentive to such matters, thereby enhancing the internal governance environment of firms’ innovation through external monitoring [21]. An excellent corporate management structure and an innovative organizational environment can effectively promote the growth of innovation research funding and enhance its impact. Conversely, the innovation climate of a company can be used as a moderating factor to adjust its degree of financialization, which further shapes the behavioural pattern of corporate innovation research funding [22]. The risk tolerance of top leaders has the potential to directly inform a firm’s long-term strategy and indirectly influence a firm’s economic performance and decision-making [20]. A firm’s risk tolerance is reflected primarily in the investment preferences held by senior managers. These preferences indicate a tendency to invest in projects that have higher risk and higher expected returns, despite the potential for longer investment cycles and uncertain outcomes [20]. The value of a company is comprised of two key elements: the fair price of its existing assets and liabilities and the value of its future potential. The latter is contingent upon the company’s risk tolerance [20]. The decision of senior management to develop new products or services with a high risk of research and development can significantly enhance the competitiveness and market position of the company [20]. A firm’s capacity for risk-taking profoundly influences its present and future development [20]. For investors, a high level of risk is often accompanied by a high level of return, which is a common phenomenon and aligns with the underlying management philosophy [20]. In corporate operations, senior managers are more inclined to take risks and assume risks for the future competitiveness of their firms; therefore, they will increase their efforts in innovation expenditures, mergers and acquisitions, and investments in specific areas of expertise to strengthen their application of innovation theories so that their firms can gain a sustainable competitive advantage in the future competitive landscape of the marketplace and further increase their innovative strength [20].

H3: Corporate governance plays a significant moderating role in the effectiveness and efficacy of the NSL in promoting corporate innovation under strong capital market regulation.

## 3. Data and methodology

### 3.1 Variable selection and data sources

This paper selects enterprise data from 2015 to 2022, with a total of 19,955 observations. Three treatments are performed: (1) excluding the financial category; (2) excluding ST and *ST enterprises; and (3) excluding enterprises with a large number of missing values. To reduce the effect of extreme values, the continuous variables in this paper are shrink-tailed in the upper and lower 1% quartiles. All company data in this paper come from the China Stock Market & Accounting Research (CSMAR) database. The descriptions of each variable are shown in Table 1, and the basic characteristics of all the variables are presented in Table 2.

**Table 1:** Description of the variables.

	VarName	Variable Name	Variable Calculation
Explanatory variable	InnoEff1	Corporate innovation effectiveness	This paper uses a measure of firms’ innovation efficiency, the formula of which is ln(number of inventions independently filed in the year + number of utility models independently filed in the year + number of designs independently filed in the year +1)/ln(1 + total R&D expenditures)
	TFP_GMM	Issues of innovation effectiveness of enterprises	Firms’ innovation effectiveness measured using total factor productivity
Core explanatory variable	inter	New securities law (NSL)	${\text{treatment}}_{i}\times {\text{post}}_{t}$
Intermediary variables	SA	Financing constraints	The financing constraints index is taken in absolute terms
	ADJ_Roa	Risk tolerance	Level of risk taking [23]
	GorGov	Corporate governance	
Control variables	Size	Enterprise size	Natural logarithm of total company assets
	Lev	Financial leverage	Total liabilities/total assets
	FIXED	Fixed assets as a percentage	Net fixed assets to total assets
	Cashflow	Cash flow ratio	Net cash flows from operating activities divided by total assets
	Growth	Revenue growth rate	(Current operating income – prior period’s revenue)/prior period’s operating income
	Board	Board size	Natural logarithm of the number of board members
	TOP1	Shareholding concentration	Number of shares held by the largest shareholder/total number of shares
	FirmAge	Years of establishment	In (current year – year of incorporation + 1)

**Table 2:** Descriptive statistical analysis of the variables.

VarName	Obs	Mean	SD	Min	Median	Max.
InnoEff1	19955	0.1732	0.0783	0.0000	0.1823	0.3342
TFP_GMM	19955	5.7021	0.7757	3.8232	5.6020	8.0873
inter	19955	0.1540	0.3610	0.0000	0.0000	1.0000
SA	19955	3.8634	0.2534	2.0936	3.8638	5.6899
ADJ_Roa	19955	−0.0127	0.0735	−0.6810	−0.0111	0.3093
EU	19955	6.3328	0.5311	5.0213	6.4722	6.8409
GorGov	19955	0.0302	0.6367	−1.7308	−0.1693	2.4064
Size	19955	22.3193	1.2542	19.8079	22.1310	26.2443
Lev	19955	0.4074	0.1912	0.0580	0.4006	0.9032
FIXED	19955	0.1976	0.1395	0.0024	0.1706	0.6875
Cashflow	19955	0.0520	0.0698	−0.6581	0.0493	0.8385
Growth	19955	0.1724	0.3725	−0.5619	0.1128	2.3571
Board	19955	2.1055	0.1953	1.6094	2.1972	2.7081
TOP1	19955	0.3266	0.1429	0.0846	0.3040	0.7400
FirmAge	19955	2.9760	0.2900	1.6094	2.9957	4.1744

### 3.2 Modelling

#### 3.2.1 DID model.

To test the theoretical hypotheses, this paper employs the implementation of the NSL as a quasinatural experiment, utilizing the DID model to assess the effect of the NSL on the issue of corporate innovation effectiveness and efficacy. The DID model allows for the testing of whether there is a significant difference between the experimental group and the control groups with respect to the issue of corporate innovation effectiveness and efficacy before and after the implementation of the NSL. According to the median division of regional administrative law enforcement intensity, the experimental group has the highest administrative law enforcement intensity, and the control group has the lowest administrative law enforcement intensity. In this paper, the experimental group comprises 1,570 enterprises, whereas the control group consists of 2,474 enterprises. Accordingly, the model is defined as follows:


$$\begin{array}{c} \text{InnoEff}1_{it}=\alpha_{10}+\alpha_{11}{\text{inter}}_{it}+\alpha_{12}\text{contro}l_{it}+\delta_{i}+\rho_{t}+\varepsilon_{it} \end{array}$$ (1)

$$\begin{array}{c} TFP\_{GMM}_{it}=\alpha_{20}+\alpha_{21}{\text{inter}}_{it}+\alpha_{23}\text{contro}l_{it}+\delta_{i}+\rho_{t}+\varepsilon_{it} \end{array}$$ (2)

The subscripts i and t denote individuals and years, respectively, and firm innovation effectiveness (InnoEff1) and the firm innovation potency problem (TFP_GMM) are the explanatory variables. ${\text{inter}}_{it}$ (${\text{inter}}_{it}$=${\text{treatment}}_{i}\times post_{t}$) is the core explanatory variable. ${\text{treatment}}_{i}$ denotes the experimental and control group dummy variables, where the experimental group variable takes a value of 1 and the control group variable takes a value of 0. $post_{t}$ is the policy year dummy variable, with the 2020 and later year variables taking a value of 1 and the previous year variables taking a value of 0. The control variables are as follows: firm size (SIZE), financial leverage (LEV), cash flow level (CASHFLOW), firm growth (GROWTH), fixed asset ratio (FIXED), equity concentration (TOP1), board size (BOARD), board of directors (BOD), board of directors (BOARD), board size (Board), and firm age (FirmAge). $\delta_{i}$ denotes individual fixed effects. $\rho_{t}$ denotes time effects, and to address potential serial correlation and heteroskedasticity, robust standard errors for individual clustering are reported in the regression results in the subsequent section.

#### 3.2.2 Mediating effects model.

To investigate the transmission mechanism or possible mediating variables of NSL on the issue of the effectiveness and efficacy of corporate innovation, this section adopts the mediating effect model, using financing constraints (SA) and risk taking (ADJ_Roa) as the mediating variables M to conduct the empirical analysis, and constructs the model as follows:


$$\begin{array}{c} \text{InnoEff}1_{it}=\alpha_{30}+\alpha_{31}{\text{inter}}_{it}+\alpha_{32}\text{contro}l_{it}+\delta_{i}+\rho_{t}+\varepsilon_{it} \end{array}$$ (3)

$$\begin{array}{c} M_{it}=\alpha_{40}+\alpha_{41}{\text{inter}}_{it}+\alpha_{42}\text{contro}l_{it}+\delta_{i}+\rho_{t}+\varepsilon_{it} \end{array}$$ (4)

$$\begin{array}{c} TFP\_{GMM}_{it}=\alpha_{50}+\alpha_{51}{\text{inter}}_{it}+\alpha_{52}\text{contro}l_{it}+\delta_{i}+\rho_{t}+\varepsilon_{it} \end{array}$$ (5)

$$\begin{array}{c} M_{it}=\alpha_{60}+\alpha_{61}{\text{inter}}_{it}+\alpha_{62}\text{contro}l_{it}+\delta_{i}+\rho_{t}+\varepsilon_{it} \end{array}$$ (6)

The above set of equations determines whether a transmission relationship exists between the “core explanatory variables - mediating variables - explanatory variables”, i.e., whether the mediating variables are important influence paths. First, the first equation for each mediating effect is similar to the fixed effects model presented in the preceding section. It measures the impact of the NSL and other control variables on the effectiveness and efficiency of corporate innovation and answers the question of whether the core explanatory variables have an impact on the explanatory variables. Second, the second formula for each mediating effect, namely, the mediating variables SA and ADJ_Roa, serves to ascertain whether there is a significant effect of the core explanatory variables on the mediating variables, answering the question of “whether the core explanatory variables have a significant effect on the mediating variables”. Ultimately, the two-step approach proposed by Jiang [24] is employed to elucidate the relationship between the mediator variables and the explanatory variables, thereby answering the question of whether the mediator variables affect the explanatory variables.

#### 3.2.3 Moderating effects.

When there is a moderating effect of the moderating variable, the coefficient estimation in the regression model should consider not only the direct effect of the independent variable on the dependent variable but also the moderating effect of the moderating variable on this relationship. Therefore, this paper constructs the moderating effect model as follows:


$$\begin{array}{c} \text{InnoEff}1_{it}=\alpha_{70}+\alpha_{71}{\text{inter}}_{it}+\alpha_{72}{\text{inter}}_{it}*W_{it}+\alpha_{73}W_{it}+\alpha_{74}\text{contro}l_{it}+\delta_{i}+\rho_{t}+\varepsilon_{it}\# \end{array}$$ (7)

$$\begin{array}{c} TFP\_GMM_{it}=\alpha_{80}+\alpha_{81}{\text{inter}}_{it}+\alpha_{82}{\text{inter}}_{it}*W_{it}+\alpha_{83}W_{it}+\alpha_{84}\text{contro}l_{it}+\delta_{i}+\rho_{t}+\varepsilon_{it}\# \end{array}$$ (8)

where W is the moderating variable, which in this paper is corporate governance (GorGov).

### 3.3 Correlation analysis

After the relevant data are obtained, these data are analysed to study the relationships among the variables. Correlation analysis is a very widely used method. It is a statistical analysis method that does not consider the causal relationship between variables but only studies and analyses the correlation between variables. Commonly used correlation analysis methods include simple correlation analysis and partial correlation analysis.

Therefore, this paper performs a basic correlation analysis on the data pertaining to enterprise innovation effectiveness (InnoEff1), TFP_GMM, NSL (inter), financing constraints (SA), risk-taking (ADJ_Roa), enterprise size (SIZE), and financial leverage (LEV). Furthermore, additional variables were considered, and the results are presented below.

As shown in Table 3, the correlation coefficient between the variables, and the results show that the correlation coefficient between the effectiveness of corporate innovation and the NSL is 0. The correlation coefficient is 0.98 and is statistically significant at the 1% level. A larger absolute value of this coefficient indicates a stronger relationship between the two variables, suggesting a relatively high degree of correlation. As a whole, the correlation coefficients of the variables are not close to −1 or 1 and are within the range of −0.5 to 0.5. This finding indicates that the variables are more independent and that the possibility of a negative impact on the subsequent regression analysis is low. The results demonstrate that the selected data are reliable overall, alleviating the covariance problem of the regression equation to a certain extent, and indicate that subsequent regression analyses can be carried out.

**Table 3:** Correlation analysis.

	InnoEff1	TFP_GMM	inter	Size	Lev	FIXED	Cashflow	Growth	Board	TOP1	FirmAge
InnoEff1	1										
TFP_GMM	0.155***	1									
inter	0.098***	0.146***	1								
Size	0.338***	0.627***	0.091***	1							
Lev	0.201***	0.409***	0.148***	0.509***	1						
FIXED	0.002	−0.268***	−0.067***	0.118***	0.084***	1					
Cashflow	0.013*	0.062***	−0.017**	0.079***	−0.166***	0.197***	1				
Growth	0.035***	0.146***	−0.012*	0.046***	0.029***	−0.045***	0.048***	1			
Board	0.094***	0.122***	0.013*	0.279***	0.137***	0.119***	0.033***	−0.007	1		
TOP1	0.023***	0.096***	−0.062***	0.158***	0.031***	0.111***	0.111***	−0.014*	0.014*	1	
FirmAge	0.012	0.145***	0.146***	0.178***	0.142***	0.044***	0.008	−0.082***	0.099***	−0.042***	1

Note: ***p < 0.01, **p < 0.05, *p < 0.1.

## 4. Empirical results

### 4.1 Benchmark regression results

The empirical analysis is conducted on the basis of panel data through the selection of indicators and model setting and testing in the above subsection, as illustrated in Table 4. In the regression process, the NSL is used as the base variable, and then other control variables are added to the regression so that the results are more stable.

**Table 4:** Main regression results.

	(1)	(2)	(3)	(4)
VarName	InnoEff1	InnoEff1	TFP_GMM	TFP_GMM
inter	0.0030**	0.0049***	0.0309***	0.0558***
	(2.210)	(3.605)	(3.360)	(7.401)
Size		0.0197***		0.2491***
		(16.706)		(38.139)
Lev		−0.0039		0.0937***
		(−0.906)		(3.961)
FIXED		0.0044		−1.7102***
		(0.699)		(−48.675)
Cashflow		−0.0128**		0.7454***
		(−2.115)		(22.204)
Growth		−0.0007		0.2227***
		(−0.759)		(42.159)
Board		0.0083**		0.0170
		(2.288)		(0.850)
TOP1		0.0137*		−0.0642
		(1.766)		(−1.493)
FirmAge		−0.0133		0.1619***
		(−1.253)		(2.759)
_cons	0.1553***	−0.2602***	5.3677***	−0.2750
	(148.029)	(−6.858)	(761.471)	(−1.310)
Firm FE	Yes	Yes	Yes	Yes
Year FE	Yes	Yes	Yes	Yes
N	19955	19955	19955	19955
R^2^	0.7656	0.7702	0.8923	0.9284

Note: ***p < 0.01, **p < 0.05, *p < 0.1.

In the estimation results presented in Table 4, Columns 1 and 2 include only the NSL as an explanatory variable for regression analysis. The regression results indicate that the NSL passes the significance test. In Column 2, after adding control variables, the estimated results indicate that this policy led to a significant increase of 0.0049 units in enterprise innovation efficiency (InnoEff1). This may be because the NSL has a positive effect on enterprise innovation efficiency. The new regulations likely provide clearer and more specific legal protections for enterprise innovation, enabling firms to invest resources in innovation with greater confidence and reduced legal risks. Moreover, the new regulations might have strengthened requirements for information disclosure, improving market transparency and helping investors and enterprises better understand the value and risks of innovation projects, thereby enhancing enterprises’ ability to secure innovation funding.

Additionally, in Column (4), after adding control variables, the estimated results show that the coefficient of the NSL on TFP_GMM is significantly 0.0558. A possible explanation is that the NSL may have optimized corporate financing channels, offering more convenient and diversified financing options, such as bond and equity markets, thereby providing a more stable source of funding for enterprise innovation activities. Furthermore, the new regulations might have strengthened investor protections, increasing market transparency and fairness, which encourages investors to invest in innovative enterprises and provides additional financial support for enterprise innovation activities.

### 4.2 Mediating effects

#### 4.2.1 Financing constraints (SA).

As Table 5 shows the regression results of financing constraints as a transmission mechanism, according to the mediation effect test step, the results in Columns (1) and (2) are consistent with the baseline regression results and will not be repeated. Column (3) presents the empirical regression results. The regression coefficient is −0.0044 and is significant at the 1% level, which suggests that the NSL will reduce financing constraints. The NSL may provide more financing channels for firms by liberalizing market access, simplifying the approval process, and lowering the financing threshold. This includes the bond market, the equity market, private placement financing, etc., which enables enterprises to raise capital more easily, thus easing financing constraints. In addition, the new regulations may have strengthened the disclosure requirements and increased the transparency of information in the market. This helps investors better understand the financial status, operational status and future development potential of enterprises, thus increasing investor confidence in enterprises and promoting capital inflows. Through the mediation effect test, financing constraints are significant as a mediating variable, and the NSL promotes the issue of corporate innovation effectiveness and efficacy by reducing financing constraints, thereby enhancing the efficacy and effectiveness of corporate innovation.

**Table 5:** Intermediation effects.

	(1)	(2)	(3)
VarName	InnoEff1	TFP_GMM	SA
inter	0.0049***	0.0558***	−0.0044***
	(3.605)	(7.401)	(−3.734)
Size	0.0197***	0.2491***	−0.0076***
	(16.706)	(38.139)	(−7.408)
Lev	−0.0039	0.0937***	0.0370***
	(−0.906)	(3.961)	(9.969)
FIXED	0.0044	−1.7102***	−0.0275***
	(0.699)	(−48.675)	(−4.994)
Cashflow	−0.0128**	0.7454***	−0.0122**
	(−2.115)	(22.204)	(−2.308)
Growth	−0.0007	0.2227***	0.0045***
	(−0.759)	(42.159)	(5.406)
Board	0.0083**	0.0170	−0.0008
	(2.288)	(0.850)	(−0.269)
TOP1	0.0137*	−0.0642	−0.0793***
	(1.766)	(−1.493)	(−11.756)
FirmAge	−0.0133	0.1619***	0.0650***
	(−1.253)	(2.759)	(7.059)
_cons	−0.2602***	−0.2750	3.7202***
	(−6.858)	(−1.310)	(112.885)
Firm FE	Yes	Yes	Yes
Year FE	Yes	Yes	Yes
N	19955	19955	19955
R^2^	0.7702	0.9284	0.9835

Note: ***p < 0.01, **p < 0.05, *p < 0.1.

#### 4.2.2 Assumption of risk (ADJ_Roa).

As Table 6 shows the regression results for risk-taking as a transmission mechanism, the results in Columns (1) and (2) are consistent with the baseline regression results according to the steps of the mediation effect test and will not be repeated. Column (3) presents the empirical regression results. The regression coefficient is 0.0081 and is significant at the 1% level, which indicates that the NSL enhances the level of corporate risk-taking. This is because the NSL provides a more stable and favourable legal environment for corporate innovation and venture capital. Enhanced legal security helps firms feel more comfortable in taking on the uncertainties associated with innovation and risky investment because they know that they will be able to obtain some protection and relief under the legal framework even if they face failure. The results of the mediation effect test demonstrate that risk-taking plays a significant role as a mediating variable. The NSL is thus shown to contribute to the issue of corporate innovation effectiveness and efficacy by enhancing the level of risk-taking and thus the effectiveness of innovation.

**Table 6:** Intermediation effects.

	(1)	(2)	(3)
VarName	InnoEff1	TFP_GMM	ADJ_Roa
inter	0.0049***	0.0558***	0.0081***
	(3.605)	(7.401)	(4.696)
Size	0.0197***	0.2491***	0.0279***
	(16.706)	(38.139)	(18.712)
Lev	−0.0039	0.0937***	−0.1961***
	(−0.906)	(3.961)	(−36.357)
FIXED	0.0044	−1.7102***	−0.0997***
	(0.699)	(−48.675)	(−12.443)
Cashflow	−0.0128**	0.7454***	0.2154***
	(−2.115)	(22.204)	(28.130)
Growth	−0.0007	0.2227***	0.0390***
	(−0.759)	(42.159)	(32.376)
Board	0.0083**	0.0170	−0.0009
	(2.288)	(0.850)	(−0.192)
TOP1	0.0137*	−0.0642	0.0686***
	(1.766)	(−1.493)	(6.993)
FirmAge	−0.0133	0.1619***	0.0009
	(−1.253)	(2.759)	(0.065)
_cons	−0.2602***	−0.2750	−0.5673***
	(−6.858)	(−1.310)	(−11.848)
Firm FE	Yes	Yes	Yes
Year FE	Yes	Yes	Yes
N	19955	19955	19955
R^2^	0.7702	0.9284	0.5852

Note: ***p < 0.01, **p < 0.05, *p < 0.1.

### 4.3 Moderating effects

As shown in Table 7, the results for Columns (1) and (3) are identical to those presented above and, as a result, will not be repeated. Column (2) shows that the coefficient of NSL (inter) is 0.0050 and is significant at the 1% level. This implies that NSL has a significant positive effect on firms’ innovation effectiveness. In other words, the implementation of the NSL increases the innovation effectiveness of firms. The coefficient of corporate governance (GorGov) is 0.0038 and is significant at the 5% level. This finding indicates that corporate governance has a significant positive effect on firms’ innovation effectiveness. In other words, good corporate governance contributes to the innovation effectiveness of firms. This is because good corporate governance provides a stable decision-making environment, encourages innovation and risk-taking, and ensures that resources for innovation activities are effectively allocated. The coefficient of the interaction term (inter_GorGov) is 0.0003 and is statistically insignificant. This finding indicates that the interaction term between the NSL (inter) and corporate governance (GorGov) has no significant effect on the effectiveness of corporate innovation. In other words, there is no significant difference in the effectiveness of the implementation of the new securities act due to differences in corporate governance. This is probably because the new securities act contains universal principles and provisions that are applicable to all firms; therefore, differences in corporate governance structures do not significantly affect its effectiveness.

**Table 7:** Moderating effects.

	(1)	(2)	(3)	(4)
VarName	InnoEff1	InnoEff1	TFP_GMM	TFP_GMM
inter	0.0049***	0.0050***	0.0558***	0.0500***
	(3.605)	(3.572)	(7.401)	(6.454)
Size	0.0197***	0.0200***	0.2491***	0.2476***
	(16.706)	(16.847)	(38.139)	(37.776)
Lev	−0.0039	−0.0040	0.0937***	0.0969***
	(−0.906)	(−0.927)	(3.961)	(4.097)
FIXED	0.0044	0.0045	−1.7102***	−1.7085***
	(0.699)	(0.708)	(−48.675)	(−48.642)
Cashflow	−0.0128**	−0.0124**	0.7454***	0.7414***
	(−2.115)	(−2.049)	(22.204)	(22.088)
Growth	−0.0007	−0.0008	0.2227***	0.2226***
	(−0.759)	(−0.786)	(42.159)	(42.162)
Board	0.0083**	0.0074**	0.0170	0.0234
	(2.288)	(2.038)	(0.850)	(1.162)
TOP1	0.0137*	0.0093	−0.0642	−0.0339
	(1.766)	(1.167)	(−1.493)	(−0.768)
FirmAge	−0.0133	−0.0113	0.1619***	0.1611***
	(−1.253)	(−1.061)	(2.759)	(2.727)
inter_GorGov		0.0003		−0.0312***
		(0.134)		(−2.933)
GorGov		0.0038**		−0.0283***
		(2.447)		(−3.333)
_cons	−0.2602***	−0.2685***	−0.2750	−0.2592
	(−6.858)	(−7.018)	(−1.310)	(−1.226)
Firm FE	Yes	Yes	Yes	Yes
Year FE	Yes	Yes	Yes	Yes
N	19955	19955	19955	19955
R^2^	0.7702	0.7703	0.9284	0.9285

Note: ***p < 0.01, **p < 0.05, *p < 0.1.

Column (4) shows the positive impact of the NSL on the effectiveness of corporate innovation. The NSL provides a more stable and favourable legal environment for corporate innovation, which enables companies to invest resources in the innovation process with more confidence and reduces legal risks. This helps firms better capitalize on market opportunities and promote their innovation projects. Moreover, the new regulations may promote the effectiveness of enterprise innovation by improving the capital market, optimizing the incentive mechanism for innovation, promoting the protection of intellectual property rights, enhancing information transparency and regulating market competition, thereby providing a more stable source of funding for enterprise innovation activities, reducing the cost of innovation, improving the legitimacy and security of innovation results, enhancing the incentive for enterprise innovation and improving the ability of enterprises to obtain funds for innovation.

However, the negative impact of corporate governance on the issue of firms’ innovation effectiveness may be because poorer corporate governance structures lead to the wasting of firms’ innovation resources, inefficient decision-making, and inadequate incentives to innovate. Poorer corporate governance structures may make it difficult for firms to effectively utilize the innovation incentives and safeguards provided by the NSL, thus affecting the effectiveness of firms’ innovation.

For the interaction term between the NSL and corporate governance, the interaction term has a significant negative effect on the issue of firms’ innovation effectiveness. This finding indicates that corporate governance has a more significant negative effect on the issue of firms’ innovation effectiveness under the influence of the NSL. This may be because a poorer corporate governance structure makes it difficult for firms to effectively utilize the innovation incentives and safeguards provided by the new regulations, thus weakening the positive impact of the new regulations on firms’ innovation. In other words, the innovation incentives and safeguards provided by the NSL may not be effectively translated into firm innovation in a poorer corporate governance environment.

### 4.4 Heterogeneity analysis

#### 4.4.1 Heterogeneity—heavy pollution or not.

As shown in Table 8, the coefficient of the innovation effectiveness of firms (InnoEff1)—heavily polluted cities: the new securities act (inter) is 0.0072 and is significant at the 5% level. This means that the innovation effectiveness of heavily polluted cities increases by 0.0072 units under the new securities act. This result indicates that the new securities act has a positive effect on innovative activities in heavily polluted cities. One potential explanation for this is that the NSL may offer enhanced financial assistance for innovative activities within enterprises, encompassing tax incentives and R&D subsidies, among other measures. This could serve to mitigate the financial burden associated with innovation, thereby fostering a greater propensity for enterprises to engage in such activities. In addition, the new regulations provide financial incentives for firms to reduce emissions by setting emission allowances and allowing trading of carbon emission rights. This prompts enterprises to reduce the use of high-carbon emission energy and switch to low-carbon emission energy, thereby promoting the green transformation of heavily polluted cities.

**Table 8:** Heterogeneity analysis—heavy pollution or not.

	(1)	(2)	(3)	(4)
VarName	InnoEff1—Heavy Pollution	InnoEff1—Nonheavy Pollution	TFP_GMM—Heavy Pollution	TFP_GMM—Nonheavy Pollution
inter	0.0072**	0.0039**	0.0658***	0.0450***
	(2.419)	(2.499)	(4.633)	(5.213)
Size	0.0204***	0.0203***	0.1787***	0.2566***
	(7.662)	(14.881)	(13.995)	(33.518)
Lev	0.0231**	−0.0099**	−0.2615***	0.2125***
	(2.473)	(−2.029)	(−5.855)	(7.800)
FIXED	−0.0176	0.0196**	−1.1409***	−1.9715***
	(−1.535)	(2.499)	(−20.827)	(−44.816)
Cashflow	0.0021	−0.0168**	0.8459***	0.6890***
	(0.162)	(−2.446)	(13.551)	(17.885)
Growth	−0.0011	−0.0005	0.2473***	0.2158***
	(−0.476)	(−0.447)	(22.652)	(35.882)
Board	0.0118	0.0075*	0.0142	0.0190
	(1.425)	(1.850)	(0.355)	(0.841)
TOP1	0.0250	0.0058	0.0754	−0.0816
	(1.589)	(0.642)	(0.999)	(−1.604)
FirmAge	−0.0493**	−0.0035	−0.0119	0.2335***
	(−2.056)	(−0.294)	(−0.104)	(3.501)
_cons	−0.2077**	−0.2920***	1.8509***	−0.6681***
	(−2.477)	(−6.709)	(4.607)	(−2.741)
Firm FE	Yes	Yes	Yes	Yes
Year FE	Yes	Yes	Yes	Yes
N	4427	15528	4427	15528
R^2^	0.7294	0.7834	0.9369	0.9310

Note: ***p < 0.01, **p < 0.05, *p < 0.1.

The coefficient of innovation effectiveness of firms (InnoEff1)—nonheavily polluted cities: the new securities act (inter) is 0.0039 and is significant at the 5% level. This implies that the innovation effectiveness of nonheavily polluted cities has increased by 0.0039 units under the new securities act. This suggests that the new securities act has positively affected innovative activities in nonheavily polluted cities as well. The possible reason for this is that the new securities act may enhance the incentives for innovation by enhancing market transparency and protecting investors’ rights. Such cities usually already have better infrastructure and fewer environmental problems, and the new law may further enhance innovation effectiveness by optimizing resource allocation and access to finance.

The coefficient of the innovation effectiveness problem of firms (TFP_GMM)—heavily polluted cities: NSL (inter) is 0.0658 and is significant at the 1% level. This means that the innovation effectiveness problem in heavily polluted cities significantly improves by 0.0658 units under the new securities act. This finding indicates that the new securities act has a significant positive effect on innovation activities in heavily polluted cities. The possible reason for this is that these cities may face greater environmental pressures and financial shortages, and the financial protection and increased transparency provided by NSL could significantly improve the innovation effectiveness of firms in these cities. Policy incentives may specifically target environmental governance and technological improvements, resulting in a significant increase in innovation effectiveness.

The coefficient of the innovation effectiveness problem of firms (TFP_GMM)—nonheavily polluted cities: NSL (inter) is 0.0450 and is significant at the 1% level. This means that the innovation effectiveness problem in nonheavily polluted cities significantly improves by 0.0450 units under the new securities act. This suggests that the new securities act has also had a significant positive effect on innovative activities in nonheavily polluted cities. The probable reason for this is that although such cities already have some innovative capacity, the implementation of NSL further improves the extent to which the innovation effectiveness problem is addressed by providing a fairer market environment and incentives. This may be because the new law introduces more technology evaluation and incentives, enabling firms to utilize their innovation resources more effectively.

#### 4.4.2 Heterogeneity—nature of enterprises.

As shown in Table 9, the coefficient of innovation effectiveness of enterprises (InnoEff1)—state-owned enterprises: NSL (inter) is 0.0016 and statistically insignificant. This suggests that NSL has little effect on the innovation effectiveness of SOEs. The possible reason for this is that SOEs usually have more stable sources of funding and resource security, and they have relatively higher investment and capacity in innovation, so NSL has less impact on their innovation effectiveness.

**Table 9:** Heterogeneity—nature of enterprises.

	(1)	(2)	(3)	(4)
VarName	InnoEff1—State-owned	InnoEff1—Nonstate	TFP_GMM—State	TFP_GMM—Nonstate
inter	0.0016	0.0066***	0.0493***	0.0491***
	(0.649)	(3.879)	(3.579)	(5.260)
Size	0.0247***	0.0185***	0.2555***	0.2407***
	(10.261)	(12.990)	(19.137)	(31.009)
Lev	0.0085	−0.0028	−0.0113	0.1681***
	(0.951)	(−0.546)	(−0.230)	(5.950)
FIXED	−0.0055	0.0125	−1.3701***	−1.8397***
	(−0.481)	(1.591)	(−21.635)	(−42.862)
Cashflow	−0.0053	−0.0153**	0.7168***	0.7697***
	(−0.465)	(−2.075)	(11.328)	(19.221)
Growth	0.0054***	−0.0036***	0.2311***	0.2155***
	(2.854)	(−3.184)	(22.148)	(34.604)
Board	0.0063	0.0090*	0.0284	0.0121
	(0.947)	(1.959)	(0.774)	(0.484)
TOP1	−0.0132	0.0182*	0.0675	−0.1799***
	(−0.974)	(1.744)	(0.897)	(−3.164)
FirmAge	0.0033	−0.0013	0.3677***	0.1310*
	(0.150)	(−0.103)	(3.006)	(1.840)
_cons	−0.4171***	−0.2672***	−0.9751**	−0.0187
	(−5.051)	(−5.854)	(−2.129)	(−0.075)
Firm FE	Yes	Yes	Yes	Yes
Year FE	Yes	Yes	Yes	Yes
N	5586	13902	5586	13902
R^2^	0.8020	0.7586	0.9427	0.9196

Note: ***p < 0.01, **p < 0.05, *p < 0.1.

The coefficient of innovation effectiveness of firms (InnoEff1) —nonstate firms: NSL (inter) is 0.0066 and is significant at the 1% level. This implies that the innovation effectiveness of nonstate firms significantly increases by 0.0066 units under the new securities act. This is because nonstate enterprises usually face greater market competition pressure, and NSL provides nonstate enterprises with more innovation resources and motivation by providing a more stable and favourable legal environment, optimizing the capital market, and improving the incentive mechanism for innovation, thus significantly increasing their innovation effectiveness.

The coefficient of the innovation effectiveness problem of firms (TFP_GMM)—state-owned enterprises: NSL (inter) is 0.0493 and is significant at the 1% level. This means that the innovation effectiveness problem of SOEs has significantly improved by 0.0493 units under the NSL. This is because the NSL provides a more favourable innovation environment for SOEs by strengthening the protection of intellectual property rights, increasing the transparency of information, and regulating market competition, prompting them to be more proactive in their innovation activities, which significantly improves the innovation effectiveness problem.

The coefficient of the innovation effectiveness problem of firms (TFP_GMM)—nonstate-owned firms: NSL (inter) is 0.0491 and is significant at the 1% level. This implies that the innovation effectiveness problem of nonstate firms significantly improved by 0.0491 units under the implementation of the new securities act. This is because the NSL, by providing a more stable and favourable legal environment, optimizing the capital market, and improving the incentive mechanism for innovation, provides nonstate enterprises with more resources and incentives to innovate, prompting them to be more proactive in their innovation activities, thus significantly improving the innovation effectiveness problem.

#### 4.4.3 Heterogeneity—overseas background or not.

As shown in Table 10, the coefficient of innovation effectiveness of firms (InnoEff1)—with overseas background: new securities act (inter) is 0.0040 and is significant at the 5% level. This implies that the innovation effectiveness of firms with overseas backgrounds increases by 0.0040 units under the implementation of the new securities act. This is because the implementation of the NSL may provide more innovation resources for firms with overseas backgrounds, including wider opportunities for international cooperation, richer platforms for technological exchange, and more flexible financing channels, which all contribute to the innovation effectiveness of these firms.

**Table 10:** Heterogeneity analysis—overseas background or not.

	(1)	(2)	(3)	(4)
VarName	InnoEff1—With Overseas Background	InnoEff1—No Overseas Background	TFP_GMM—With Overseas Background	TFP_GMM—No Overseas Background
inter	0.0040**	0.0059**	0.0424***	0.0647***
	(2.041)	(2.550)	(3.872)	(5.251)
Size	0.0206***	0.0206***	0.1913***	0.2726***
	(11.468)	(10.468)	(19.126)	(26.109)
Lev	−0.0026	−0.0019	0.1083***	0.1093***
	(−0.417)	(−0.272)	(3.104)	(2.905)
FIXED	0.0072	0.0074	−1.6478***	−1.6758***
	(0.759)	(0.722)	(−31.004)	(−30.719)
Cashflow	−0.0081	−0.0173*	0.6556***	0.6696***
	(−0.926)	(−1.779)	(13.386)	(13.041)
Growth	−0.0025*	0.0011	0.2168***	0.2295***
	(−1.921)	(0.708)	(29.359)	(27.295)
Board	0.0161***	−0.0030	0.0005	0.0466
	(3.073)	(−0.479)	(0.015)	(1.404)
TOP1	0.0225*	0.0214*	−0.2269***	0.1977***
	(1.859)	(1.716)	(−3.370)	(2.991)
FirmAge	−0.0091	−0.0171	0.1273	0.2908***
	(−0.571)	(−0.932)	(1.427)	(2.988)
_cons	−0.3128***	−0.2479***	1.1893***	−1.3364***
	(−5.497)	(−3.753)	(3.751)	(−3.822)
Firm FE	Yes	Yes	Yes	Yes
Year FE	Yes	Yes	Yes	Yes
N	10184	9771	10184	9771
R^2^	0.8145	0.8041	0.9408	0.9456

Note: ***p < 0.01, **p < 0.05, *p < 0.1.

The coefficient of innovation effectiveness of firms (InnoEff1)—no overseas background: new securities act (inter) is 0.0059 and is significant at the 5% level. This implies that the innovation effectiveness of firms with no overseas background increases by 0.0059 units under the new securities act. This is because the NSL may provide more innovation opportunities and incentives for firms without an overseas background by optimizing innovation incentives, improving information transparency, and regulating market competition, which promotes the innovation activities of these firms.

The coefficient of the innovation effectiveness problem of firms (TFP_GMM)—with an overseas background: new securities act (inter) is 0.0424 and is significant at the 1% level. This implies that the innovation effectiveness problem of firms with overseas backgrounds significantly improves by 0.0424 units under the new securities act. This is because the NSL may have created a better innovation environment for firms with overseas backgrounds by providing a more stable and favourable legal environment, strengthening the protection of intellectual property rights, and regulating market competition, which has prompted these firms to be more proactive in their innovation activities, thus significantly improving the innovation effectiveness problem.

The coefficient of the innovation effectiveness problem of firms (TFP_GMM)—with no overseas background: new securities act (inter) is 0.0647 and is significant at the 1% level. This implies that the innovation effectiveness problem of firms with no overseas background significantly improves by 0.0647 units under the new securities act. This is because the NSL may significantly improve the innovation effectiveness problem by providing a more stable and favourable legal environment, optimizing the capital market, and improving the innovation incentive mechanism, which provides more innovation resources and motivation for firms without overseas backgrounds and prompts these firms to be more proactive in their innovation activities.

#### 4.4.4 Heterogeneity—sublife cycle.

As shown in Table 11, innovation effectiveness of firms (InnoEff1)—growing firms: the coefficient of the new securities act (inter) is 0.0067 and is significant at the 1% level. This means that the innovation effectiveness of growth firms has significantly increased by 0.0067 units under the new securities act. This is because the NSL provides a more stable and favourable legal environment for firms to innovate, which enables them to invest their resources in the innovation process with more confidence and reduce legal risk. Moreover, the new regulations may provide a more stable source of funding for enterprise innovation activities, reduce innovation costs, improve the legitimacy and security of innovation results, enhance the motivation to innovate and improve the ability to obtain funds for enterprise innovation by improving the capital market, optimizing the incentive mechanism for innovation, and promoting the protection of intellectual property rights, thus facilitating the enhancement of the effectiveness of innovation in growth-oriented enterprises.

**Table 11:** Heterogeneity—sublife cycle.

	(1)	(2)	(3)	(4)	(5)	(6)
VarName	InnoEff1—Growth	InnoEff1—Mature	InnoEff1—Recession	TFP_GMM—Growth	TFP_GMM—Mature	TFP_GMM—Recession
inter	0.0067***	0.0035	0.0086**	0.0483***	0.0734***	0.0275
	(2.795)	(1.385)	(2.015)	(3.698)	(6.604)	(1.020)
Size	0.0189***	0.0162***	0.0266***	0.2089***	0.3147***	0.2448***
	(9.379)	(6.122)	(6.696)	(18.954)	(26.701)	(9.723)
Lev	0.0024	−0.0189**	0.0126	0.0613	0.0049	0.2250***
	(0.336)	(−2.061)	(0.960)	(1.582)	(0.121)	(2.707)
FIXED	0.0229**	−0.0095	0.0375	−1.7016***	−1.5701***	−2.3723***
	(2.139)	(−0.790)	(1.626)	(−29.087)	(−29.149)	(−16.257)
Cashflow	−0.0253**	−0.0168	−0.0135	0.5609***	0.8981***	0.4646***
	(−2.121)	(−1.236)	(−0.789)	(8.612)	(14.823)	(4.276)
Growth	−0.0026*	−0.0007	0.0025	0.1924***	0.2237***	0.2943***
	(−1.692)	(−0.321)	(0.857)	(23.333)	(23.066)	(15.749)
Board	0.0017	0.0106	0.0116	−0.0006	−0.0001	0.1084
	(0.272)	(1.552)	(1.081)	(−0.016)	(−0.003)	(1.595)
TOP1	−0.0114	0.0238	0.0317	−0.1781**	−0.1999***	−0.2904*
	(−0.833)	(1.580)	(1.264)	(−2.392)	(−2.975)	(−1.830)
FirmAge	0.0060	−0.0026	−0.0025	0.2097**	0.0486	0.2510
	(0.334)	(−0.130)	(−0.070)	(2.151)	(0.539)	(1.102)
_cons	−0.2721***	−0.2154***	−0.4654***	0.5287	−1.2543***	−0.4622
	(−4.323)	(−2.706)	(−3.506)	(1.540)	(−3.534)	(−0.550)
Firm FE	Yes	Yes	Yes	Yes	Yes	Yes
Year FE	Yes	Yes	Yes	Yes	Yes	Yes
N	8251	7589	4030	8251	7589	4030
R^2^	0.8208	0.8412	0.8626	0.9485	0.9656	0.9460

Note: ***p < 0.01, **p < 0.05, *p < 0.1.

Innovation effectiveness of firms (InnoEff1)—mature firms: the coefficient of NSL (inter) is 0.0035, but it is not significant. This may be because mature firms usually already have a more mature management system and a stable business model, so the impact of the NSL on their innovation effectiveness is relatively small.

The coefficient of innovation effectiveness of firms (InnoEff1)—declining firms: new securities act (inter) is 0.0086 and is significant at the 5% level. This implies that the innovation effectiveness of declining firms increases by 0.0086 units under the new securities act. This may be because the NSL provides declining firms with opportunities for transformation and rebirth through the provision of legal safeguards, optimization of financing channels, and incentives to innovate, thus increasing their innovation effectiveness.

The coefficient of the innovation effectiveness problem of firms (TFP_GMM)—growth firms: NSL (inter) is 0.0483 and is significant at the 1% level. This implies that the innovation effectiveness problem of growing firms significantly improves by 0.0483 units under the new securities act. This may be because the implementation of the NSL has provided more innovation resources and incentives for growing firms, including more flexible financing channels, richer platforms for technology exchange, and stronger legal protections, which have helped improve the innovation efficiency and effectiveness of these firms.

The coefficient of the innovation effectiveness problem of firms (TFP_GMM)—mature firms: NSL (inter) is 0.0734 and is significant at the 1% level. This implies that the innovation effectiveness problem of mature firms significantly improves by 0.0734 units under the implementation of the new securities act. This may be because the implementation of the NSL provides more innovation resources and incentives for mature firms, including more flexible financing channels, richer technology exchange platforms, and stronger legal protections, which all contribute to improving the innovation efficiency and effectiveness of these firms.

The coefficient of the firms’ innovation effectiveness problem (TFP_GMM)—declining firms: NSL (inter) is 0.0275 but not significant. This may be because declining firms face more complex challenges and problems, and the new securities act, while providing some assistance, may not be sufficient to significantly improve their innovation effectiveness problems in the short term.

#### 4.4.5 Heterogeneity—Competition in submarkets.

As shown in Table 12, in the innovation effectiveness of enterprises (InnoEff1)—highly competitive environment, the NSL provides a fairer and more regulated market environment for enterprises in highly competitive environments by strengthening market transparency and protection mechanisms. This helps firms better cope with market competition and reduce unnecessary legal risks, which leads to a 0.0034 unit increase in innovation effectiveness. However, as enterprises in high-competition markets face greater competitive pressures and resource constraints, the effect of the new law is relatively small, and the increase in the innovation effectiveness of enterprises is also relatively small.

**Table 12:** Heterogeneity analysis—competition in submarkets.

	(1)	(2)	(3)	(4)
VarName	InnoEff1—High Competition	InnoEff1—Low Competition	TFP_GMM—High Competition	TFP_GMM—Low Competition
inter	0.0034*	0.0082***	0.0472***	0.0716***
	(1.929)	(3.358)	(4.702)	(5.943)
Size	0.0191***	0.0195***	0.2283***	0.2774***
	(12.142)	(8.357)	(25.475)	(23.945)
Lev	−0.0037	−0.0046	0.0998***	0.0494
	(−0.677)	(−0.554)	(3.237)	(1.199)
FIXED	0.0279***	−0.0282***	−1.8247***	−1.5221***
	(3.252)	(−2.692)	(−37.322)	(−29.306)
Cashflow	−0.0183**	−0.0042	0.6434***	0.8213***
	(−2.334)	(−0.420)	(14.410)	(16.588)
Growth	−0.0006	−0.0014	0.2316***	0.2125***
	(−0.470)	(−0.835)	(33.272)	(26.079)
Board	0.0150***	0.0082	0.0130	0.0383
	(3.280)	(1.260)	(0.498)	(1.192)
TOP1	−0.0167	0.0363**	−0.0809	0.0638
	(−1.139)	(2.395)	(−0.967)	(0.851)
FirmAge	−0.0031	−0.0365**	0.1447*	0.0215
	(−0.222)	(−1.981)	(1.789)	(0.235)
_cons	−0.2832***	−0.2024***	0.2226	−0.5742
	(−5.541)	(−2.858)	(0.763)	(−1.637)
Firm FE	Yes	Yes	Yes	Yes
Year FE	Yes	Yes	Yes	Yes
N	12078	7877	12078	7877
R^2^	0.7687	0.8066	0.9228	0.9518

Note: ***p < 0.01, **p < 0.05, *p < 0.1.

In terms of firms’ innovation effectiveness (InnoEff1) —in low competition environments, the NSL improves innovation effectiveness by 0.0082 units by enabling firms in low-competition environments to focus more on innovation activities through increased market transparency and the provision of more innovation resources and support. In less competitive environments, the transparency and incentives brought about by the new law can significantly enhance firms’ ability to innovate, as firms have more time and resources to devote to innovative activities.

In terms of firms’ innovation effectiveness (TFP_GMM)—high competitive environment, the NSL significantly improves the problem of firms’ innovation effectiveness by 0.0472 units. The impact of an improved legal environment on firms’ innovation effectiveness is more significant in a highly competitive market because competition itself pushes firms to focus more on efficiency and innovation. By providing more legal safeguards and incentives, the new law helps firms better cope with market competition, thus improving their innovation effectiveness.

In terms of firms’ innovation effectiveness (TFP_GMM)—low competition environment, the NSL leads to a greater improvement in the innovation effectiveness problem of firms in low-competition environments by 0.0716 units through the provision of additional innovation resources and support. Since firms in low-competitive environments face less pressure to innovate, the new act leads to a greater improvement in the innovation effectiveness problem of these firms by providing additional incentives and resources. This suggests that the effect of the new law is more significant in low-competitive environments, as firms have more time and resources to devote to innovation activities.

### 4.5 Inspection

#### 4.5.1 Parallel trend test.

**Fig 1:**
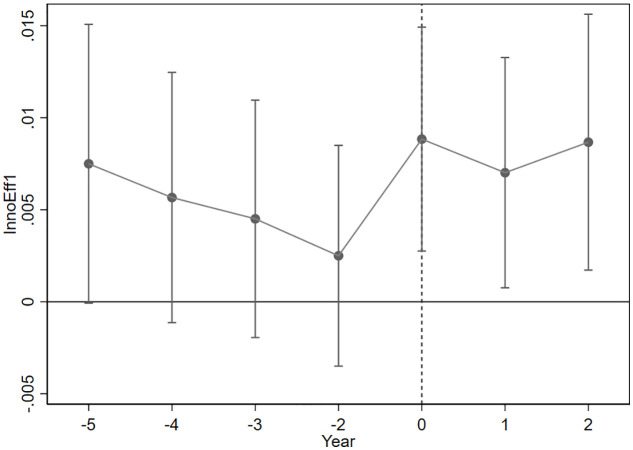
InnoEff1 Parallel trend test.

**Fig 2:**
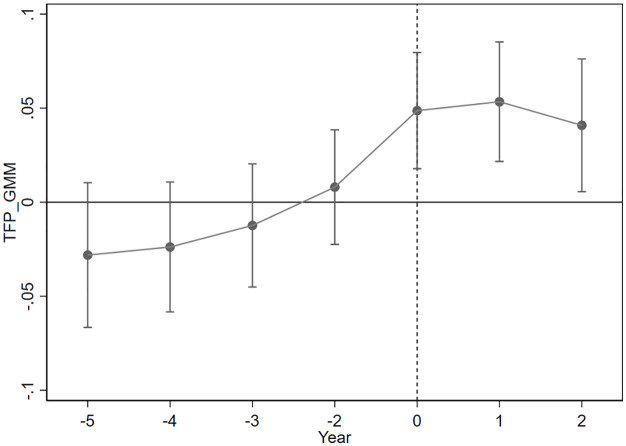
TFP_GMM Parallel trend test.

A dynamic effects test essentially introduces a finite number of time dummy variables and cross-multiplies them with the treatment group dummy variables to examine the significance of the cross-multiplication term. There is a difference between the dynamic effects test and the parallel trend test. In the parallel trend test, it is sufficient to examine whether the cross-multiplication term is significant before period 0. If it is not significant, it means that there is no significant difference between the treatment group and the control group beforehand, and the DID can be used. The interaction terms between the dummy variables and the treatment group dummy variables are first generated, and these interaction terms are regressed as the explanatory variables. The coefficients of the interaction terms reflect the specific differences between the treatment and control groups. As shown in Figs 1 and 2, the coefficients of the interaction terms are not significantly different from 0 before the policy (the 95% confidence interval includes a value of 0), indicating that there is no significant difference between the treatment and control groups at the prepolicy time point. This observation aligns with the assumption of parallel trends.

#### 4.5.2 Placebo test.

**Fig 3:**
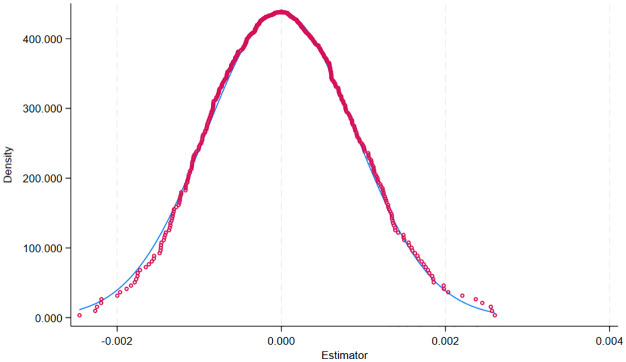
InnoEff1 Placebo test for coefficient distribution.

**Fig 4:**
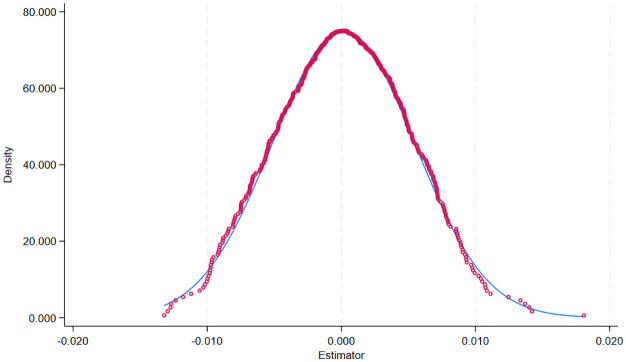
TFP_GMM Placebo test for coefficient distribution.

In the placebo test, the most common approach is the individual placebo test, which involves randomly selecting individuals as the treatment group and repeating this process 500 or 1000 times to assess the significance of the coefficient of the “pseudopolicy dummy variable.” Therefore, to test whether the empirical results of the double-difference model constructed in the previous section are robust and to ensure the rigor of the research results, 500 regression results (including the estimated coefficients of the “pseudopolicy dummy variables”) are obtained by repeating the process 500 times. The standard error and the p value are obtained, and finally, the results of the 500 regressions are plotted. The distribution of the estimated coefficients of the “pseudopolicy dummy variables” can be plotted to visualize the results of the placebo test, and the results are shown in Figs 3 and 4. The distribution of the coefficients also clearly shows that the randomly sampled coefficients are normally distributed with zero as the mean. This suggests that the estimates are unlikely to have been obtained by chance and thus are unlikely to have been influenced by other policy or randomness factors. Thus, the model passes the placebo test.

#### 4.5.3 Robustness tests.

(1) Replacement of the regression method for PSM-DID

The idea behind using propensity score matching is to measure the probability of an individual entering the experimental group because it is very difficult to compare and match multiple observable but uncontrollable eigenvalues. To find a control sample of individuals in the experimental group, factors other than the independent variable can be made as similar as possible, i.e., matched, and the multidimensional covariates can be replaced by a single one-dimensional variable so that the two are comparable and the probability that an individual will enter the experimental group can be determined by the propensity score of the individual. It would then only be necessary to match on a single propensity score variable so that differences in the explanatory variables can be observed through differences in that variable. This approach was first proposed by Rosenbaum & Rubin [25].

As shown in Table 13, the test results reveal that the significance and positive and negative correlations of the core explanatory variables also do not change significantly, which proves that the previously established model is reasonable and that the regression results are stable. Notably, the significance and positive and negative correlations of the other control variables did not change significantly, indicating that the previously established model is reasonable and that the regression results are stable and reliable.

**Table 13:** Robustness test results—PSM-DID.

	(1)	(2)	(1)	(2)
VarName	InnoEff1	InnoEff1	TFP_GMM	TFP_GMM
inter	0.0036**	0.0054***	0.0175*	0.0463***
	(2.374)	(3.630)	(1.778)	(5.697)
Size		0.0195***		0.2456***
		(14.868)		(35.110)
Lev		−0.0001		0.0742***
		(−0.022)		(2.911)
FIXED		0.0081		−1.6627***
		(1.144)		(−43.561)
Cashflow		−0.0161**		0.7607***
		(−2.369)		(20.755)
Growth		−0.0019*		0.2222***
		(−1.791)		(38.492)
Board		0.0091**		−0.0176
		(2.259)		(−0.820)
TOP1		0.0145*		−0.0007
		(1.695)		(−1.445)
FirmAge		−0.0026		0.1664***
		(−0.221)		(2.597)
_cons	0.1570***	−0.2887***	5.3957***	−0.1323
	(138.993)	(−6.845)	(738.974)	(−0.579)
Firm FE	Yes	Yes	Yes	Yes
Year FE	Yes	Yes	Yes	Yes
N	16428	16428	17768	17768
R^2^	0.7734	0.7779	0.8952	0.9293

Note: ***p < 0.01, **p < 0.05, *p < 0.1.

(2) Replacement of the explanatory variables

Replacing the explanatory variables is a common robustness test when performing regression analyses aimed at reducing estimation bias due to unobserved variables. By replacing the explanatory variables, the reliability and accuracy of the regression results are thus improved. The regression results are presented in the table.

As shown in Table 14, the significance and positive and negative correlations of the variables have not changed significantly, indicating that the previously developed model is reasonable and that the regression results are stable and reliable. This means that the modelling carried out on the issue of the effectiveness and efficacy affecting corporate innovation is reliable and that the interpretation and analysis of these factors is accurate and valid.

**Table 14:** Robustness of replacing the explanatory variables.

	(1)	(2)
VarName	Replacement variable—InnoEff2	Substitution of variables—TFP_LP
inter	0.0060***	0.0589***
	(3.836)	(7.749)
Size	0.0198***	0.4754***
	(14.532)	(72.214)
Lev	−0.0035	0.1337***
	(−0.719)	(5.607)
FIXED	0.0031	−1.4033***
	(0.429)	(−39.626)
Cashflow	−0.0142**	0.7618***
	(−2.032)	(22.513)
Growth	−0.0009	0.2323***
	(−0.851)	(43.625)
Board	0.0099**	0.0517**
	(2.366)	(2.559)
TOP1	0.0134	−0.0094
	(1.501)	(−0.217)
FirmAge	−0.0175	0.2646***
	(−1.433)	(4.474)
_cons	−0.2124***	−3.0599***
	(−4.859)	(−14.462)
Firm FE	Yes	Yes
Year FE	Yes	Yes
N	19955	19955
R^2^	0.7478	0.9574

Note: ***p < 0.01, **p < 0.05, *p < 0.1.

(3) Excluding municipalities

In the regression analysis, municipalities directly under the central government may differ from other cities in some aspects because of their special political status and resource endowment. Such differences may lead to the endogeneity problem, i.e., the policy effects may be influenced by the characteristics of the municipality itself rather than by the policy implementation; thus, the regression results are shown in the table.

As shown in Table 15, the results of the robustness test further consolidate the conclusions drawn from the previous empirical analysis. Specifically, the robustness test shows that the significance of the core explanatory variables and their positive correlation with the effectiveness and efficacy of firms’ innovations have not changed significantly, which is a good indication that the stability of the regression results is quite high.

**Table 15:** Robustness of municipality removal.

	(1)	(2)
VarName	Municipalities—InnoEff1	Municipalities—TFP_GMM
inter	0.0062***	0.0526***
	(4.083)	(6.396)
Size	0.0200***	0.2525***
	(15.388)	(35.925)
Lev	−0.0039	0.0863***
	(−0.816)	(3.367)
FIXED	0.0041	−1.6559***
	(0.598)	(−44.208)
Cashflow	−0.0109	0.7119***
	(−1.616)	(19.446)
Growth	−0.0009	0.2114***
	(−0.853)	(36.620)
Board	0.0069*	0.0045
	(1.680)	(0.202)
TOP1	0.0148*	−0.0186
	(1.742)	(−0.405)
FirmAge	−0.0118	0.1227*
	(−0.982)	(1.891)
_cons	−0.2657***	−0.2609
	(−6.267)	(−1.136)
Firm FE	Yes	Yes
Year FE	Yes	Yes
N	16208	16208
R^2^	0.7576	0.9270

Note: ***p < 0.01, **p < 0.05, *p < 0.1.

(4) Removing the impact of the epidemic

The impact of the epidemic should be excluded owing to the fact that when the analysis is conducted within the scope of the entire dataset, different data segments can often be calculated in a way that the conclusions obtained may be completely different. Therefore, to test whether the empirical results presented in the previous section are robust and ensure the rigor of the research results, this paper conducts a robustness test by excluding the epidemic data (2020), and the results are shown in the table below.

As shown in Table 16, the results of the significance test for the core explanatory variables show that they are still statistically significant and that their positive correlations maintain their original direction, which indicates that the model setting and estimation methods in the previous section are appropriate and effective in capturing the true relationships among the variables.

**Table 16:** Robustness tests excluding the effect of the epidemic.

	(1)	(2)
VarName	Culling the Epidemic—InnoEff1	Culling Outbreaks—TFP_GMM
inter	0.0058***	0.0529***
	(3.663)	(5.967)
Size	0.0199***	0.2532***
	(15.766)	(35.875)
Lev	−0.0042	0.0967***
	(−0.902)	(3.740)
FIXED	0.0065	−1.7355***
	(0.944)	(−44.976)
Cashflow	−0.0121*	0.7368***
	(−1.796)	(19.662)
Growth	−0.0003	0.2167***
	(−0.331)	(36.948)
Board	0.0077*	0.0204
	(1.948)	(0.924)
TOP1	0.0124	−0.0826*
	(1.476)	(−1.766)
FirmAge	−0.0133	0.1485**
	(−1.179)	(2.358)
_cons	−0.2638***	−0.3245
	(−6.514)	(−1.435)
Firm FE	Yes	Yes
Year FE	Yes	Yes
N	17431	17431
R^2^	0.7703	0.9270

Note: ***p < 0.01, **p < 0.05, *p < 0.1.

## 5. Conclusion

Studies have shown that in markets characterized by uncertainty, well-regulated environmental policies can promote corporate innovation [26,27]. The establishment of the NSL enhances the quality of corporate audits and facilitates high-quality corporate development [28]. As financial market uncertainties have become increasingly pronounced, particularly since the 2008 financial crisis, developed countries such as the United States, Germany, and the United Kingdom have undertaken securities regulation reforms to increase investor protection and promote stock market development [29,30]. Although China’s securities market reforms began relatively late, they have garnered substantial attention. Drawing on data from Chinese listed firms, our study supports the notion that optimizing institutional environments, particularly improving capital market policies, plays a critical role in fostering corporate innovation. This conclusion aligns with the majority of the literature examining the relationship between institutional environments and corporate innovation [31–33].

Our research elucidates the potential mechanisms through which the NSL influences corporate innovation, namely, by alleviating financing constraints, enhancing risk-taking capacity, and strengthening corporate governance. First, while previous studies have not reached a consensus on whether stringent securities market regulation constrains corporate financing [29,34], we find that the implementation of China’s NSL improves information transparency and market norms, enabling firms to secure financing at lower costs and attract broader investor interest. This finding aligns with Daouk et al. (2006) [35], who demonstrated that securities laws aimed at improving capital market governance effectively reduce equity costs and increase market liquidity, thereby easing corporate financing constraints. Second, consistent with the majority of studies, we observe that securities laws enhance investor protection and reduce insider trading, thereby attracting more long-term investors to the market. A diversified investor structure mitigates market risk, which in turn increases firms’ risk-taking capacity [36,37]. Finally, regarding the impact of securities regulation on corporate governance, our findings support Mallin et al. (2005) [38], who argued that efficient and reliable capital markets are critical for ensuring corporate governance quality. Similarly, La Porta, Lopez-de-Silanes [39] found that robust investor protection significantly enhances internal governance quality.

Furthermore, our study also reveals that the corporate governance environment effectively moderates the positive impact of NSL on firms’ innovation, as evidenced by the strengthened positive association between the two. As Pistor, Keinan [40] emphasized, corporate governance significantly influences how institutional factors, such as corporate law, affect firm performance and innovation. Similarly, our findings align with those of Chen and Hsu [41], who showed that sound corporate governance structures, such as higher board independence, amplify firms’ innovation efforts under external regulatory oversight.

This paper examines the impact of the NSL from the perspectives of the effectiveness and efficiency of corporate innovation, aiming to provide evidence for advancing capital market reforms and improving corporate governance. On the basis of the previous analysis, a regression model was constructed, with the recently enacted Securities Law serving as the primary explanatory variable and combining it with corporate innovation effectiveness as the explanatory variable from a macro perspective while considering the relevant control variables. The empirical analysis of the model yielded the following key conclusions. First, the NSL has a significant positive effect on the issue of corporate innovation effectiveness and validity. This suggests that the implementation of new regulations helps improve the innovation ability and innovation effectiveness of enterprises, thus promoting their innovation activities. This may be because the new regulations provide a more stable and favourable legal environment for firms to innovate, which enables them to invest their resources in the innovation process with more confidence and reduce legal risks. Moreover, the new regulations may, by improving the capital market, optimizing the incentive mechanism for innovation, promoting the protection of intellectual property rights, improving the transparency of information, and regulating market competition, provide a more stable source of funding for enterprises’ innovation activities, reduce the cost of innovation, improve the legitimacy and safety of innovation results, enhance the motivation of enterprises to innovate, and improve the ability to obtain funds for innovation, thus facilitating the effectiveness of enterprises’ innovation and improving the problem of innovation effectiveness.

Second, the impact of the NSL on the effectiveness and efficacy of corporate innovation is mediated by financing constraints and risk-taking. This suggests that the new regulation promotes innovation activities by improving the financing environment of enterprises, reducing financing costs, and increasing financial support for their innovation activities. Moreover, the new regulations improve the risk-bearing capacity of enterprises by providing legal safeguards and strengthening the protection of intellectual property rights, which makes enterprises more willing to invest resources in innovation activities.

Finally, corporate governance plays an important moderating role in the impact of the NSL on the effectiveness of corporate innovation. This suggests that a good corporate governance structure contributes to the effectiveness of the implementation of the new regulations and improves the efficiency and effectiveness of firms’ innovation activities. Conversely, inadequate corporate governance structures may impede the effectiveness of the implementation of the new regulations and impact the development of firms’ innovation activities.

Therefore, the following policy recommendations are proposed. First, optimizing the financing environment is key to promoting corporate innovation. The government should simplify the financing process and reduce financing costs, providing more financing channels and financial support to enterprises, particularly small and medium-sized enterprises (SMEs) and startups. This will not only help enterprises secure sufficient funding but also enhance their innovation capabilities and reduce the financial risks associated with innovation activities. Additionally, further strengthening intellectual property protection is crucial. The government should increase legal penalties for intellectual property infringements to ensure that enterprises’ innovations are effectively protected. This will greatly increase enterprises’ motivation to innovate and promote the transformation and application of their technological innovations and products.

Furthermore, improving enterprises’ risk-bearing capacity is also an important factor in driving innovation. The government should provide more comprehensive legal protection and incentive mechanisms to strengthen enterprise confidence, enabling enterprises to take on greater innovation risks. Moreover, the government should encourage enterprises to establish robust internal risk management systems to improve their ability to respond to market changes, ensuring that enterprises can manage the uncertainties associated with innovation activities.

Finally, optimizing corporate governance structures also plays a significant role. In particular, for state-owned enterprises, the government should push forwards the reform of corporate governance structures to increase decision-making efficiency and innovation execution. Moreover, the government needs to strengthen its supervision of enterprises to ensure that they operate in compliance with regulations and protect investors’ rights. Good corporate governance will ensure the smooth implementation of innovation policies and improve the efficiency of corporate innovation.

## Supporting information

S1 File data2. (DTA)

S2 File CODE. (DO)
